# Non-Noble Metal Oxide Catalysts for Methane Catalytic Combustion: Sonochemical Synthesis and Characterisation

**DOI:** 10.3390/nano7070174

**Published:** 2017-07-07

**Authors:** Przemysław J. Jodłowski, Roman J. Jędrzejczyk, Damian K. Chlebda, Anna Dziedzicka, Łukasz Kuterasiński, Anna Gancarczyk, Maciej Sitarz

**Affiliations:** 1 Faculty of Chemical Engineering and Technology, Cracow University of Technology, Warszawska 24, 31-155 Kraków, Poland; akjdz@poczta.fm; 2 Malopolska Centre of Biotechnology, Jagiellonian University, Gronostajowa 7A, 30-387 Kraków, Poland; roman.jedrzejczyk@uj.edu.pl; 3 Faculty of Chemistry, Jagiellonian University, Ingardena 3, 30-060 Kraków, Poland; damian.chlebda@uj.edu.pl; 4 Jerzy Haber Institute of Catalysis and Surface Chemistry, Polish Academy of Sciences, Niezapominajek 8, 30-239 Kraków, Poland; nckutera@cyf-kr.edu.pl; 5 Institute of Chemical Engineering, Polish Academy of Sciences, Bałtycka 5, 44-100 Gliwice, Poland; anna.g@iich.gliwice.pl; 6 Faculty of Materials Science and Ceramics, AGH University of Science and Technology, al. Mickiewicza 30, 30-059 Kraków, Poland; msitarz@agh.edu.pl

**Keywords:** sonochemistry, methane catalytic combustion, nanoparticles, non-noble metals

## Abstract

The aim of this study was to obtain nanocrystalline mixed metal-oxide–ZrO_2_ catalysts via a sonochemically-induced preparation method. The effect of a stabiliser’s addition on the catalyst parameters was investigated by several characterisation methods including X-ray Diffraction (XRD), nitrogen adsorption, X-ray fluorescence (XRF), scanning electron microscopy (SEM) equipped with energy dispersive X-ray spectrometer (EDS), transmission electron microscopy (TEM) and µRaman. The sonochemical preparation method allowed us to manufacture the catalysts with uniformly dispersed metal-oxide nanoparticles at the support surface. The catalytic activity was tested in a methane combustion reaction. The activity of the catalysts prepared by the sonochemical method was higher than that of the reference catalysts prepared by the incipient wetness method without ultrasonic irradiation. The cobalt and chromium mixed zirconia catalysts revealed their high activities, which are comparable with those presented in the literature.

## 1. Introduction

Nanostructured transition metal oxides, due to their specific magnetic, optical, and catalytic properties, have been under vigorous investigation over the years [1,2,3,4]. Nanoparticles have been used in many applications, from energy storage to medical and environmental applications [5,6,7,8,9]. From a wide range of possible applications of nanoparticles, catalysis seems to be the most beneficial and exploited field. For many years of the application of nanoparticles, much effort has been directed towards the synthesis and characterisation of highly functionalised nanostructured materials that would conform to the size and structure requirements for catalytic processes. The development of preparation techniques such as chemical reduction, sputtering, chemical dealloying, microwave assisted synthesis, and deposition methods (Chemical Vapour Deposition, Physical Vapour Deposition), along with the possibility of preparing highly-defined catalyst nanoparticles, have been the subject of increased research in the field of catalysis [2]. The synthesis of well-defined catalytic structures with specific shapes and morphologic parameters has become a kind of scientific art, in which nanostructures can be created in the form of three-dimensional nano-flowers, two-dimensional nano-plates, and one-dimensional nano-rods or nano-belts [10]. From a wide group of nanomaterials, those based on d-block elements are of special interest in the field of catalysis. There are various methods of nanoparticle preparation, but the sonochemically-assisted method has shown great potential for obtaining well-defined nanoparticles for catalytic purposes [11,12,13,14,15,16,17,18].

Ultrasounds passing through the solutions causes cavitation, which refers to the growth and collapse of bubbles present in a liquid phase. Two types of sonochemical reactions may be mentioned: homogenous sonochemistry, resulting from the formation of radicals (or their intermediates), and heterogeneous sonochemistry, determined by the mechanical effects of cavitation (surface cleaning, particle size reduction, and improved mass transfer) [19]. The cavitation is generated as a result of the periodic expansion and compression of the liquid phase. The expansion of an ultrasonic field causes a negative pressure, since the gas cannot be dissolved in liquid anymore, and the bubbles are created. During compression, the opposite effect occurs: the gas within the bubble diffuses back into liquid. However, not all of the gas diffusing into the bubble diffuses out, due to a smaller surface area. After many cycles of expansion and compression, bubbles reach a critical size, resulting in their collapse during one compression cycle. Collapse of these bubbles causes the quasi-adiabatic heating of gas inside the bubble. These cavitations are responsible for the production of radicals initiating sonochemical reactions. The maximum temperature in these “hot points” reaches 5000 K, whereas the local pressure can go up to 1000 atmospheres, with a high heating/cooling rate of ca. 10^10^ K/s [19]. The occurrence of such reaction conditions leads to the bond breaking in the sonicated solutions, which results in an initiation of a series of chemical reactions (e.g., reduction of metal ions present in the irradiated solution). The simultaneous bubble collapse results in a noticeable temperature decrease and a prevention of particle agglomeration [11].

The mechanism of the particle growth as a result of the sonochemical irradiation may differ depending on the solutions used. However, the overall process of the mechanism can be described as follows [11]:

H_2_O → H* + OH* (1)

H* + H* → H_2_ (2)

H* + OH* → H_2_O (3)

OH* + OH* → H_2_O_2_ (4)

RH (volatile or nonvolatile vapours) → R* + H*(5)

RH + OH* (or H*) → R* + H_2_O (6)

M*^n^*^+^ (Co^2+^, Cu^2+^, Cr^2+^, Pd^2+^) + *n*R* → M^0^ (7)

*m*M^0^ → M*_m_*. (8)

According to the literature reports, the use of the volatile vapors during the sonication results in the formation of amorphous nanomaterials, whereas the use of nonvolatile vapours (e.g., SDS) favours the formation of well-defined crystalline structures [19].

However, even though sonochemistry can be a simple and versatile tool for the preparation of catalysts with optimised nanoparticle parameters such as morphology, shape, and structure, the optimisation of the preparation method is very important due to the strong dependency of those parameters on the synthesis conditions. For example, the discontinuous irradiation of the carrier/active metal by ultrasound can result in the synthesised materials having different properties [20]. Moreover, the application of ultrasonic irradiation during nanoparticle synthesis avoids the problems connected with the use of methods that require high pressures, elevated temperatures, and vacuum conditions, which the conventional methods have to deal with [12].

One of the possible applications of sonically prepared catalysts is their use for gas abatement purposes, especially the removal of hazardous gases from biogas-fuelled turbines or the homogeneous combustion of methane to produce energy [21]. Although the catalytic removal of CH_4_, NO*_x_*, CO, and Volatile Organic Compounds (VOC) from flue gases has been under vigorous investigation [22,23,24,25], the main problems connected with the choice of catalytic material, the type of reactor, and the elimination of the expensive noble-metal based catalysts remain unsolved. The literature provides several examples that can be used as alternatives to noble metal. The catalysts containing single transition metal oxides such as chromium [26], manganese [27], cobalt [28,29,30], and copper [31], as well as composite catalysts such as cobalt-palladium [32] or cobalt-chromium [26] have demonstrated superior activity in methane or VOC combustion. However, in most cited references, the catalysts are prepared either via an impregnation or a precipitation/co-precipitation method. The application of classical preparation leads to catalysts with big metal-oxide clusters at the surface. Some classical routes for preparing catalysts can be used for the synthesis of well-defined shapes and sizes of nanoparticles, but in the case of the sonochemical approach the control of the system is effortless, especially for sophisticated nanostructured reactors. The use of classical methods for the preparation of catalysts for catalytic reactions that are sensitive to catalyst crystal size and shape are less justifiable than the protocol presented here, especially in light of the catalytic activity.

In this study, we present the sonochemical approach for the preparation of nanocrystalline catalysts for methane catalytic combustion purposes.

## 2. Results and Discussion

Detailed information about catalyst preparation by the sonochemical route, and reference catalysts prepared by incipient wetness, is presented in Table 1 and Table 2, respectively. Two series of catalysts were prepared, differentiated by the addition of an SDS stabilising agent. It can be seen from the XRF results (Table 1) that the catalysts’ loading is not correlated with the addition of sodium lauryl sulfate (SDS). As an example, for the catalyst pairs Pd/ZrO_2_ and Pd/ZrO_2_/SDS, a decrease in the palladium loading can be observed; whereas for Co/ZrO_2_ and Co/ZrO_2_/SDS, the cobalt loading increases almost tenfold. On the other hand, for the copper and chromium catalyst samples, a considerable increase in the metal loading can be noticed in the case of the addition of the stabilising agent. It is also worth mentioning that the composition of the palladium catalyst (Pd/ZrO_2_) is close to those found in the literature [33] for palladium supported on alumina systems. For non-noble metal-based catalysts containing Co, Cu, or Cr as an active phase, the metal oxide loading is considerably lower than that presented in the literature studies. The typical metal loading varies from 1 to 15 wt % for cobalt impregnated zirconia catalysts [34], 0.25–9 wt % for Cu/ZrO_2_ [35], and 1.76–10.36 wt % for Cr/Al_2_O_3_ [11]. The incipient wetness method was applied to image the concentration of the sonicated catalysts. The results of specific surface areas and total pore volumes determined by nitrogen adsorption show a decrease of S_BET_ for all prepared samples when compared with the ZrO_2_ support.

The as-received zirconia support’s specific surface area is equal to 29.6 m^2^/g, whereas the specific surface area for the catalyst samples decreases and achieves the values 22.93–25.11 m^2^/g (cf. Table 1). A similar fact can be noticed when comparing the total pore volume results. The total pore volume measured for the zirconia support is equal to 0.16 cm^3^/g, whereas for the catalyst samples it decreases considerably to 0.11 cm^3^/g. The slight decrease in both S_BET_ and V_p_ are within the experimental errors. On the other hand, a considerable decrease in pore volumes can be observed for the catalyst samples prepared with the addition of SDS. However, since the overall effectiveness of the sonochemical irradiation cannot be directly measured (some of the residual ions can be still present in the catalyst precursor), the partial impregnation of the ZrO_2_ by the catalyst precursor cannot be excluded. The most significant effect is visible for the Co/ZrO_2_/SDS sample, with a V_p_ equal to 0.11 cm^3^/g. For the other prepared samples with an addition of SDS, the average pore volumes are equal 0.12, 0.13, and 0.14 for Pd/ZrO_2_/SDS, Cu/ZrO_2_/SDS, and Cr/ZrO_2_/SDS, respectively. The crystallite sizes of the prepared catalyst grains suspended in the solution, determined by dynamic light scattering (DLS), are presented in Table 1. The correlation between the particle size and the addition of an SDS stabiliser at the preparation step can be observed. The tendency towards increased particle sizes can be observed for catalysts prepared with the addition of SDS. However, this specific feature was not observed for the catalyst pairs Cu/ZrO_2_ and Cu/ZrO_2_/SDS, where the measured values were 356 ± 71 and 201 ± 80 nm, respectively. On the other hand, when considering the experimental errors, the measured values of the nanoparticle sizes did not reveal statistically significant differences. The measurements did not suggest further interaction between the ZrO_2_ support and the catalyst precursors during the calcination procedure. The influence of the calcination procedure on particle size is well known, and has been widely reported in the literature [36,37].

**Figure 1:**
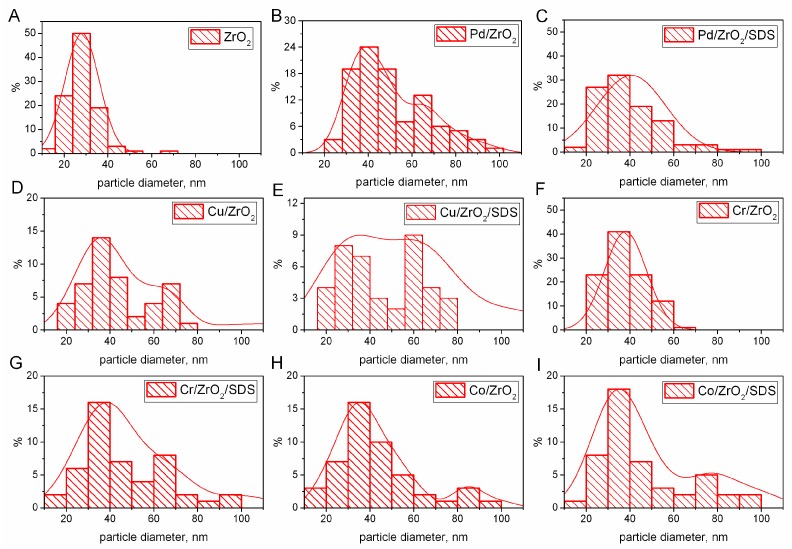
Particle size distribution histograms from analysis of TEM micrographs: (**A**) ZrO_2_; (**B**) Pd/ZrO_2_; (**C**) Pd/ZrO_2_/SDS; (**D**) Cu/ZrO_2_; (**E**) Cu/ZrO_2_/SDS; (**F**) Cr/ZrO_2_; (**G**) Cr/ZrO_2_/SDS; (**H**) Co/ZrO_2_; (**I**) Co/ZrO_2_/SDS; corresponding TEM micrographs presented in Supplementary Materials in Figure S1.

The results of the DLS measurements are, however, influenced by several factors, such as the type of solvent, the scattering contrast, and the intensity of the laser [38]. Moreover, nanoparticle size is influenced considerably by the addition of stabiliser and the thickness of the electrical double layer [39]. Despite the differences in the size of the grains in the solution and at the catalyst’s surface, this measurement is necessary to optimise the nanoparticles (NP’s) formation process. This most certainly does mean that the obtained NPs at the catalyst’s surface must be smaller due to the lack of a solvation complex. Thus, the more precise size determination has to be performed using other techniques. The result of the analysis of the TEM micrographs are presented in the form of histograms of particle size distribution in Figure 1, whereas the TEM micrographs themselves are presented in the Supplementary Materials in Figure S1.

It can be seen that the crystallite sizes determined by TEM microscopy are considerably lower than the values obtained by the DLS method. The TEM image of the untreated ZrO_2_ support was presented as a reference (Figure 1A). It can be found that the zirconia particles have a characteristic regular sphere-like structure, with a particle size range of 20–40 nm (Figure S1A). When comparing the particle size histograms for the sonically prepared catalysts and zirconia, the resulting MeO (Pd, Cu, Cr, Co) nanoparticles can be distinguished. In all of the considered catalysts, the characteristic ZrO_2_ particles in a range of 20–40 nm can be observed. For the Pd/ZrO_2_ (Figure 1B) catalysts, the additional particles can be observed in a range 55–75 nm. However, for the palladium catalysts with an SDS addition, the palladium nanoparticle sizes are lower, and 40–60 nm fractions can be observed. For copper catalysts, the sonication results in 50–80 nm particles, whereas its counterpart obtained with the addition of SDS results in the decrease of particle sizes to 40–70 nm. For the chromium catalyst (Figure 1F), an additional particle in a range of 40–60 nm can be observed, whereas for the Cr/ZrO_2_/SDS, the increase of particle sizes to 50–80 nm can be observed. For the cobalt catalysts, the sonochemical preparation without an addition of SDS results in 70–90 nm particles, whereas the addition of SDS during the sonochemical irradiation results in the decrease of particle sizes to 60–80 nm. The analysis of the particle size distribution based on the TEM results leads to the conclusion that the addition of SDS during the sonochemical preparation results in the decrease of active metal particle sizes. The phenomenon of the decrease of the particle sizes in the sonochemical preparation of nanoparticles was previously reported elsewhere [40].

**Figure 2:**
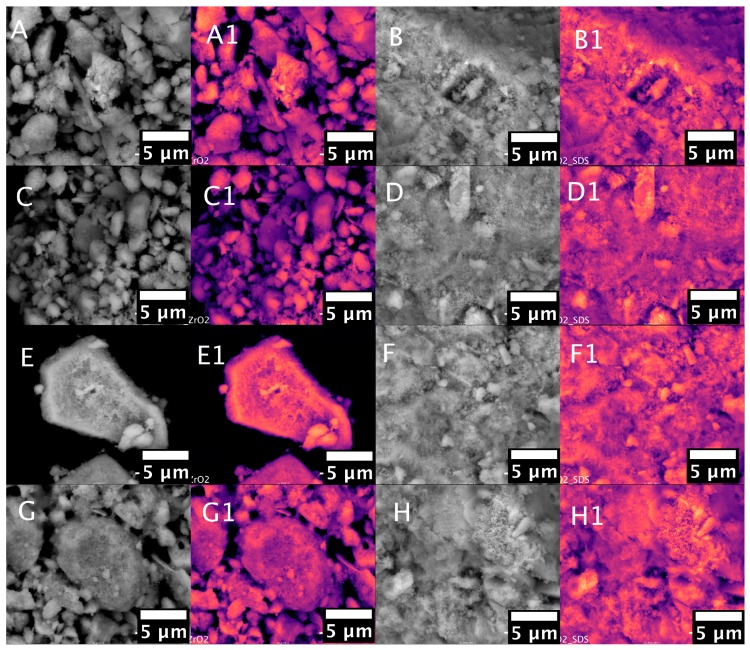
SEM micrographs of prepared catalyst samples: (**A**) Pd/ZrO_2_; (**B**) Pd/ZrO_2_/SDS; (**C**) Co/ZrO_2_; (**D**) Co/ZrO_2_/SDS; (**E**) Cu/ZrO_2_; (**F**) Cu/ZrO_2_/SDS; (**G**) Cr/ZrO_2_; (**H**) Cr/ZrO_2_/SDS; suffix 1 refers to coloured SEM.

The results of surface morphology determined by SEM microscopy are presented in Figure 2A–H.

**Figure 3:**
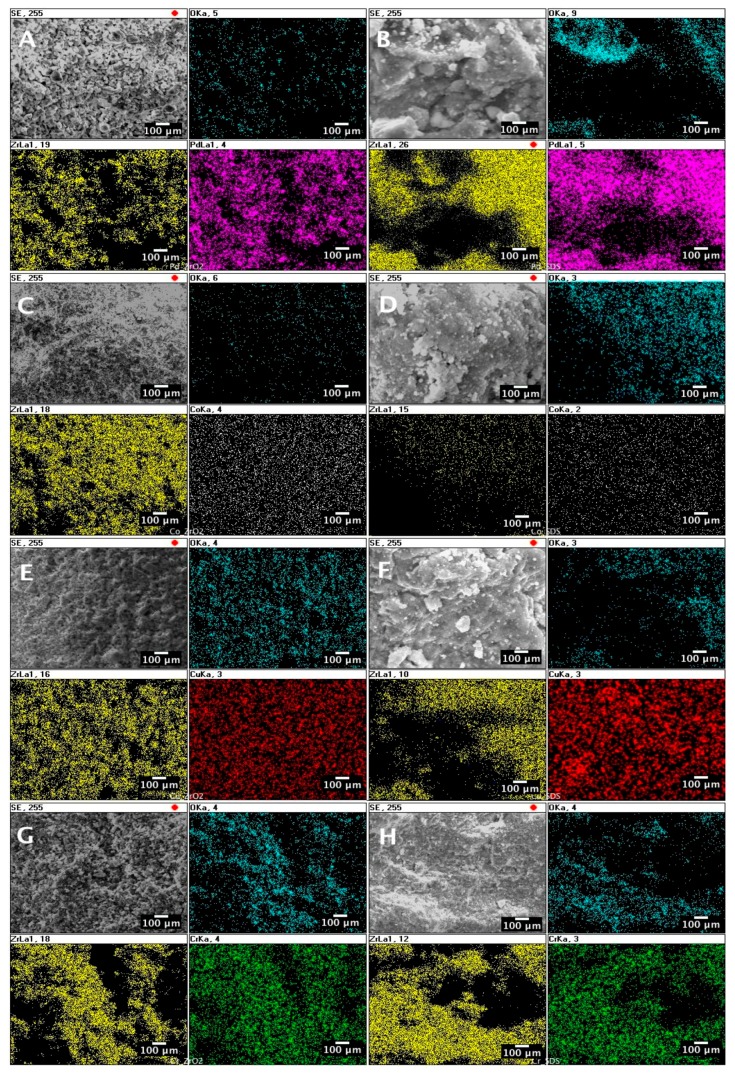
SEM/EDS mapping of prepared catalyst samples: (**A**) Pd/ZrO_2_; (**B**) Pd/ZrO_2_/SDS; (**C**) Co/ZrO_2_; (**D**) Co/ZrO_2_/SDS; (**E**) Cu/ZrO_2_; (**F**) Cu/ZrO_2_/SDS; (**G**) Cr/ZrO_2_; (**H**) Cr/ZrO_2_/SDS; Correspondence of colours and elements: O: cyan, Zr: yellow, Pd: magenta, Co: white, Cu: red, Cr: green.

To enhance the differences between the catalyst support and the deposited nanoparticles, the SEM images were taken in backscattered electron mode (BSE). Under these conditions, the differences in contrast between the nanoparticles and the catalyst support can be observed. High density materials in the BSE SEM image can be observed as light fields, whereas low density materials exhibit a low backscattered contrast [41]. To enhance this specific feature, classic BSE images were improved by using pseudocolour conversion using lookup tables (LUT) in Fiji software [42]. The procedure of SEM greyscale conversion is described in the Supplementary Material. The pseudocolour conversion is commonly performed for greyscale SEM images in, for example, the geological sciences [41]. Since the human eye cannot distinguish between grayscale colours, pseudocolouring can be performed to enhance the grey scale’s distinction. However, as can be seen from the standard SEM images (Figure 2A–H), the single nanoparticle agglomerates can be observed as a brighter spot. This specific feature is enhanced for Pd/ZrO_2_, Pd/ZrO_2_/SDS, Co/ZrO_2_/SDS, and Cu/ZrO_2_/SDS. The pseudocolour conversion of the greyscale images enhances this feature even more. In Figure 2A1,B1,D1,F1, representing the pseudocoloured SEM images of the Pd/ZrO_2_, Pd/ZrO_2_/SDS, Co/ZrO_2_/SDS, and Cu/ZrO_2_/SDS catalysts, the bright nanoparticles can be observed as intensive yellow spots. The exact determination of the nanoparticle metal distribution was performed by EDS mapping (Figure 3).

Figure 3 shows the results of the EDS mapping of the catalysts prepared by sonication with or without the addition of SDS (cf. Table 1). Figure 3 indicates the spatial distribution of the individual elements, O, Zr, and metal (Pd, Co, Cu, and Cr) impregnated on the Zr support. It can be seen from the EDS maps that, in all of the considered catalyst samples (cf. Figure 3A–H), the impregnated metals are uniformly distributed over the zirconia support. However, it can be also interfered that, for the Pd and Cu catalyst samples (Figure 3A,B,E,F), the metal impregnated is presented in an aggregated form. At the same time, individual spots of metals can be observed for the catalysts impregnated with the Co and Cr sonicated solutions (Figure 3C,D,G,H).

**Figure 4:**
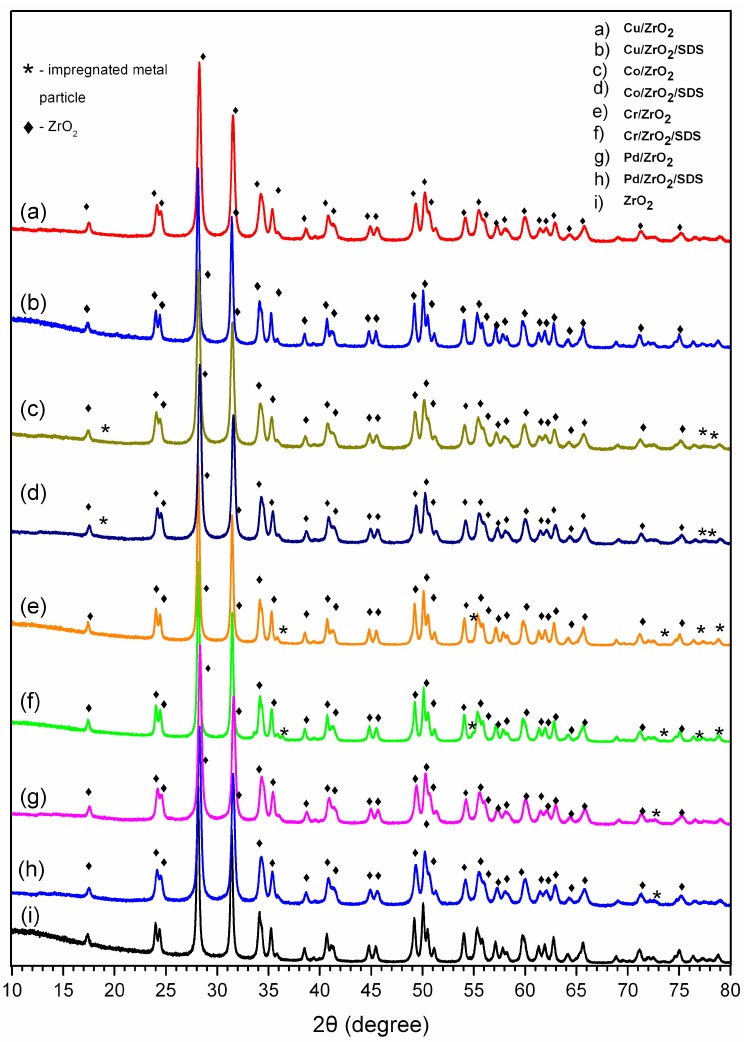
XRD patterns of prepared catalyst samples.

The XRD patterns collected for catalysts prepared via the sonochemically aided method are presented in Figure 4.

In all of the presented XRD patterns, the reflection originating from the zirconia supports can be distinguished (JCPDS 96-230-0297) (cf. Figure 4i). The ZrO_2_ reflections are denoted by rhomb symbols in all of the diffractograms (Figure 4a–h). Since the catalyst samples were calcined at 500 °C for 6 h, the analysis of the reflection patterns was limited to metal-oxide structures such as CuO, Cu_2_O, CoO, Co_3_O_4_, Cr_2_O_3_, and PdO. The reflection fitting was performed using the American Mineralogist crystal structure database [43]. The reflection originating from impregnated nanoparticle precursors was denoted by a star symbol (Figure 4a–h). It can be seen that, in most of the considered catalyst samples, the characteristic reflections for the impregnated catalysts overlap with the reflections originating from the zirconia support. However, the singular characteristic reflections can be distinguished. For the copper-based catalysts Cu/ZrO_2_ and Cu/ZrO_2_/SDS, the distinction of the characteristic reflections from CuO and Cu_2_O is not possible because the reflections overlap with the reflections from the zirconia support. An analysis of the diffractograms for the Co/ZrO_2_ and Co/ZrO_2_/SDS (Figure 4c,d) samples allows the distinction of a weak reflection at 18.9° 2θ, originating from Co_3_O_4_. The reflection is enhanced for the Co/ZrO_2_/SDS sample, due to the relatively high cobalt content (2.06 wt %) compared to Co/ZrO_2_ (0.020 wt %). The additional two reflections at 77.3° and 78.3° 2θ, originating from Co_3_O_4_, can be observed. The diffractograms for the Cr/ZrO_2_ and Cr/ZrO_2_/SDS (Figure 4e,f) catalysts reveal the characteristic reflections for the Cr_2_O_3_ structure appearing at 36.2°, 54.9°, 73.3°, 76.8°, and 79.1° 2θ. For the palladium-supported zirconia catalysts Pd/ZrO_2_ and Pd/ZrO_2_/SDS, the reflections appearing in the range of 70°–73° 2θ can be attributed to the palladium oxide structure. An in-depth analysis of the reflection patterns and a comparison with those found in the literature [24,32,44,45] cannot confirm with certainty the proposed oxide structures in the prepared catalyst samples. Since the characteristic reflections strongly overlap those originating from the zirconia support, an additional µRaman analysis of the catalysts’ morphology was performed.

**Figure 5:**
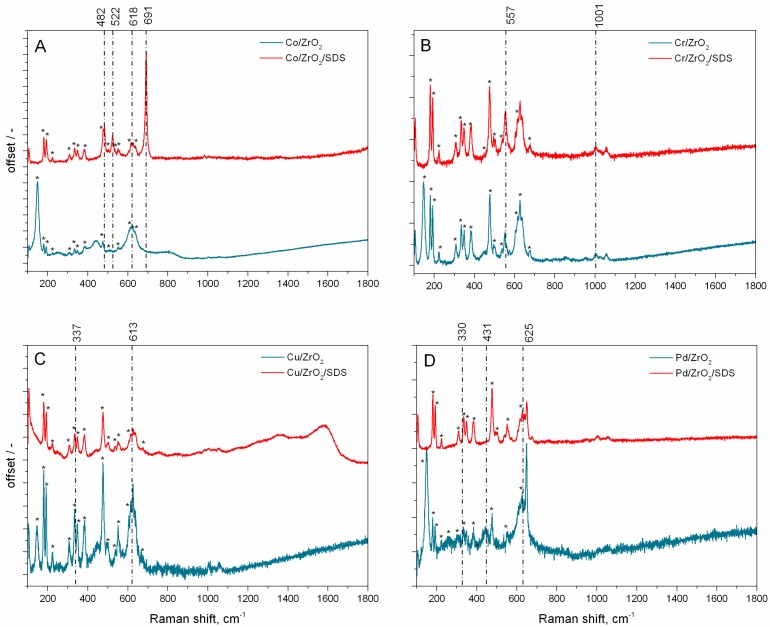
In situ μRaman analysis of sonically-prepared catalysts: (**A**) Co/ZrO_2_, Co/ZrO_2_/SDS; (**B**) Cr/ZrO_2_, Cr/ZrO_2_/SDS; (**C**) Cu/ZrO_2_, Cu/ZrO_2_/SDS; (**D**) Pd/ZrO_2_, Pd/ZrO_2_/SDS.

The results of in situ µRaman analysis are presented in Figure 5. In all presented spectra, the presence of the zirconia support was confirmed by the characteristic bands at 222, 258, 290, 326, 335, 410, 472, 553, 610, and 636 cm^−1^. The multiplicity of the Raman bands of the zirconia support confirms the existence of two phases: monoclinic and tetragonal, of ZrO_2_. The bands at 222, 335, and 472 cm^−1^ are characteristic of monoclinic ZrO_2_, whereas the bands at 258, 290, 326, 410, 553, 610, and 636 cm^−1^ can be attributed to the tetragonal phase [45,46]. The bands from the zirconia support were denoted by star symbols in all µRaman spectra (Figure 5A–D). An analysis of the Raman spectra of the Co/ZrO_2_/SDS catalyst sample (Figure 5A) reveals the bands at 482, 522, 618, and 691 cm^−1^ that can be attributed to the E_g_, F_2g_, F_2g_, and A_1g_ active modes of Co_3_O_4_, respectively [47,48]. Surprisingly, for the Co/ZrO_2_ catalyst sample, the Raman spectra reveal only the band at 618 cm^−1^, without other characteristic Co_3_O_4_ bands. The weak Raman scattering of the Co/ZrO_2_ sample can be related to the metal loading, which is 100 times lower than in the Co/ZrO_2_/SDS catalyst sample. The Raman spectra of the chromium-based samples reveal two bands at 557 and 1001 cm^−1^. The band at 557 cm^−1^ can be attributed to the A_1g_ mode of Cr_2_O_3_ [49], whereas the band at 1001 cm^−1^ can be attributed to the surface Cr=O species [50]. A closer look at the 800–900 cm^−1^ region in Cr/ZrO_2_ reveals a weak band at 856 cm^−1^ that can be associated with the polymeric O–Cr–O surface species [51]. This specific feature excludes the isolated nature of chromium species at the zirconia support for Cr/ZrO_2_ [51]. On the other hand, the O–Cr–O polymeric species for the Cr/ZrO_2_/SDS sample was not detected. However, since the spectra in this region are highly noisy, the existence of the O–Cr–O polymeric species cannot be excluded. The Raman spectrum of the copper-supported zirconia catalyst (Figure 5C) reveals two bands at 337 and 613 cm^−1^, characteristic for the B_g_ modes of CuO species [52]. An additional broad band appearing at 1130 cm^−1^ can be attributed to multi-phonon transition [52]. The multi-phonon transition can be distinguished for both the Cu/ZrO_2_ and Cu/ZrO_2_/SDS catalysts, although this band is enhanced for the Cu/ZrO_2_/SDS sample. This can be attributed to the almost double copper loading in the Cu/ZrO_2_/SDS sample, and can also be induced by local electronic density changes [52]. The high anisotropic effect may additionally be induced by the irregular morphologies of copper nanostructures [52]. The Raman analysis of the Pd/ZrO_2_ and Pd/ZrO_2_/SDS samples reveals three bands at 330, 431, and 625 cm^−1^. The bands at 431 and 625 cm^−1^ can be attributed to the E_g_ and B_1g_ characteristic modes of PdO, whereas the band at 330 and weak additional bands in the range of 1000–1300 cm^−1^ are due to a second order scattering and/or resonance effect [53].

**Figure 6:**
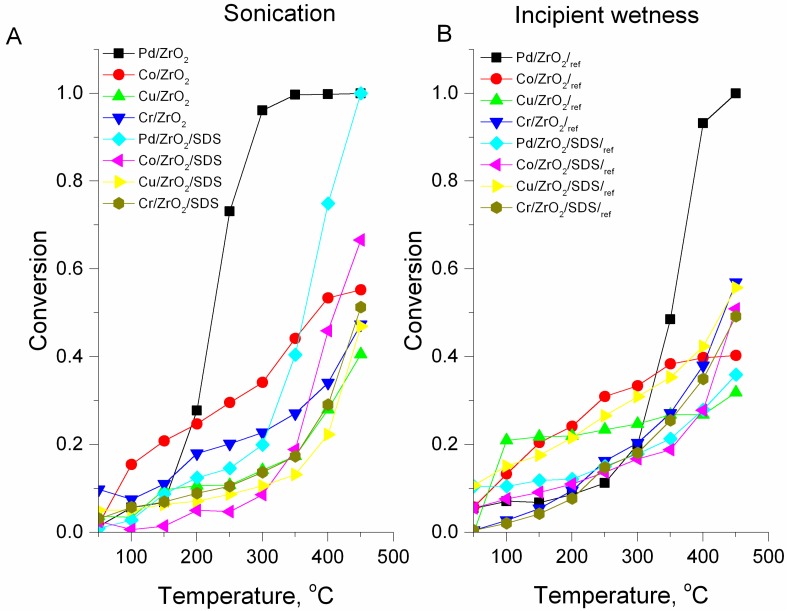
Methane catalytic combustion test results: (**A**) catalysts prepared via the sonochemical route; (**B**) reference catalysts prepared via the incipient wetness method.

The results of the catalysts’ activity in a methane catalytic combustion reaction are presented in Figure 6. The activity of the catalysts prepared via the sonochemical route (Figure 6A) were compared with their counterparts prepared via the incipient wetness method (Figure 6B). Outstanding activity was obtained by both of the palladium catalyst samples Pd/ZrO_2_ and Pd/ZrO_2_/SDS. However, the Pd/ZrO_2_ catalyst reached complete conversion at 350 °C with a light-off temperature at 225 °C, whereas the palladium catalyst obtained with an SDS addition reached complete conversion at 450 °C with T_50%_ at 360 °C. The outstanding activity of palladium catalysts is not surprising, since palladium-based catalysts are commonly used for methane catalytic combustion [54]. The activity of palladium-supported zirconia catalysts was previously reported [55] to be higher than that of alumina-supported palladium catalysts. In this study, the considerable differences in activity for both palladium-based catalysts are due to differences in palladium loading (cf. Table 1, metal content Pd/ZrO_2_ and Pd/ZrO_2_/SDS). However, when comparing the activity of Pd/ZrO_2_ and Pd/ZrO_2_/SDS with their counterparts Pd/ZrO_2_/_ref_ and Pd/ZrO_2_/SDS/_ref_, the maximum conversion was obtained only by Pd/ZrO_2_/_ref_, whereas Pd/ZrO_2_/SDS/_ref_’s activity was equal to 0.38 at 450 °C.

When comparing the light of temperatures, the T_50%_ for Pd/ZrO_2_/_ref_ was close to that for the Pd/ZrO_2_/SDS prepared via the sonochemical route and equal to 350 °C. The differences between the activities of the catalysts prepared via the sonochemical method and those obtained using incipient wetness can be related to the differences in particle sizes of the active metals impregnated. The considerable impact of particle size on the activity of the catalyst was previously reported in the literature [33,55,56]. Catalyst preparation via the incipient wetness method may favour further sintering of the metals impregnated over the zirconia support, and as a result decrease the catalyst’s activity.

From the non-noble metal catalysts, the highest activity was observed for Co/ZrO_2_/SDS obtained via the sonochemical method. The maximum conversion was approximately 70% at 450 °C (Figure 6A). For the reference sample, the maximum conversion was approx. 15% lower (Figure 6B). The remarkable activity of cobalt-based catalysts has been widely reported in the literature [24,34,57]. In work presented by Xiao et al. [34], the activity of 1 wt % Co/ZrO_2_ was tested in 1 vol % CH_4_/Air catalytic combustion. Complete conversion was achieved at 550 °C, with T_50%_ at 475 °C. The activity could be compared with that obtained in the paper published by Xiao [34], if not for the fact that, in our study, the cobalt loading is 50 times lower and is equal to 0.020 wt %. On the other hand, the notable activity of Co/ZrO_2_ in this study can be correlated with smaller catalyst particle sizes or with the cobalt distribution over the zirconia support. Indeed, the influence of crystal size on the reaction rate was previously reported in the literature [58,59,60,61], although there is still no agreement on the structure sensitivity of methane catalytic combustion. The remarkable activity of the cobalt catalysts was also correlated with the presence of cobalt in spinel form, Co_3_O_4_ [31]. In both of the considered cobalt catalysts, the presence of Co_3_O_4_ at the catalyst’s surface was confirmed by XRD (Figure 4c,d) and Raman spectroscopy (Figure 5A).

From the non-noble metal catalysts, high activity was also observed for Cr/ZrO_2_/SDS. A comparison of the activity of methane combustion over the chromium-based catalysts with the literature [11,26] reveals the high activity of the catalysts obtained in this study. In our previous study [11], we reported high activity for a sonically-prepared 10 wt % Cr_2_O_3_/Al_2_O_3_ catalyst in methane combustion. Complete activity was achieved at 500 °C. However, in this study, we report on zirconia-supported catalysts with strongly decreased chromium content (0.23 wt % for Cr/ZrO_2_ and 1.44 wt % for Cr/ZrO_2_/SDS; see Table 1). In the literature [24,26], the activity was confirmed for bulk Cr_2_O_3_ and mixed cobalt-chromium catalysts, respectively. However, Paredes et al. [24] found that the combustion of 2000 ppm CH_4_/Air mixture resulted in 45% methane conversion at 450 °C, whereas in the work published by Chen et al. [26] the conversion was below 20%. The activity of the reference chromium catalysts, Cr/ZrO_2_/_ref_ and Cr/ZrO_2_/SDS/_ref_, was close to those prepared by the sonication method, but the overall activity expressed by the trends of the light-off curves was lower. When comparing the activity of the reference Cr/ZrO_2_/_ref_ catalysts (Figure 6B) with the Cr/ZrO_2_ catalyst prepared by the sonochemical route, it can be seen that the sonically-prepared catalyst is more active at lower temperatures.

The copper-based catalysts revealed the lowest activity in methane catalytic combustion. The maximum conversion was reached by the Cu/ZrO_2_ catalysts, equal to 40% at 450 °C, whereas for the catalysts prepared by an SDS addition, the maximum conversion was 44% at 450 °C. However, the reference catalysts prepared by incipient wetness revealed the opposite tendency. The activity of Cu/ZrO_2_/SDS/_ref_ was slightly higher than its counterpart, achieving a conversion of approx. 55% at 450 °C, whereas the maximum conversion for Cu/ZrO_2_ at that temperature was 30%.

Since the catalysts considerably vary in their active metal loading, the activity of methane catalytic combustion at a maximum of 450 °C was also presented in a form of specific activity related to the active metal loading. The results are presented in Table 1 and Table 2. When considering the specific activity of the catalysts prepared via the sonochemical method related to the active metal loading, the activity towards the methane combustion decreases in the following order: Co/ZrO_2_ > Pd/ZrO_2_/SDS > Cr/ZrO_2_ > Cu/ZrO_2_ > Cu/ZrO_2_/SDS > Cr/ZrO_2_/SDS > Co/ZrO_2_/SDS > Pd/ZrO_2_. Similar trends can be observed for the reference catalysts (cf. Table 2). However, in the case of the reference catalysts, the lowest activity was observed for the Co/ZrO_2_/SDS/_ref_ catalyst sample. When comparing the results of the specific activity for both catalyst series (Table 1 and Table 2) with the average catalyst particle size, a direct correlation cannot be observed. However, an increase in specific activity can be noticed for Co/ZrO_2_ and Pd/ZrO_2_/SDS, with the decreasing of the average particle size (80 and 50 nm, respectively). This phenomenon was not observed for the other catalyst samples. Although the increase in activity of the catalysts in methane catalytic combustion with the increase of the particle sizes was previously reported for noble and non-noble based metal-oxide catalysts [33], the direct correlation between catalysts prepared via the sonochemical method with particle sizes would require the unification of the catalysts’ loading. On the other hand, the comparison of the specific activity between the catalysts prepared via the sonication and incipient methods leads to the conclusion that catalysts prepared via the sonication method have considerably higher activity in methane catalytic combustion.

## 3. Materials and Methods

### 3.1. Materials

The Pd(NO_3_)_2_·2H_2_O, Co(NO_3_)_2_·6H_2_O, Cr(NO_3_)_3_·9H_2_O and Cu(NO_3_)_2_·3H_2_O, and C_12_H_25_SO_4_Na (SDS) were supplied by Sigma Aldrich (Saint Louis, MO, USA), and the ZrO_2_ was supplied by Alfa Aesar (Haverhill, MA, USA). All of the chemicals were used as received, and employed without further purification.

### 3.2. Catalyst Preparation

In this study, mixed metal-oxide–ZrO_2_ catalysts for methane catalytic combustion were prepared by impregnation using sonochemically irradiated catalyst precursors (Pd, Co, Cu, and Cr) with or without the addition of sodium dodecyl sulphate (SDS). At the first step of the synthesis, the catalyst precursors were dissolved in deionised water to obtain the appropriate solutions, which are specified in Table 1. To obtain the nanoparticle suspensions, the dissolved catalyst precursors were sonochemically irradiated for 1 h using a QSonica S-4000 sonicator (Church Hill Rd, Newtown, CT, USA) equipped with a ½″ diameter horn (the average electrical power of sonication equals 90 W and frequency 20 kHz). During the sonication procedure, the glass tube filled with the catalyst precursor with or without SDS (0.17% *w*/*v*) was placed in an ice bath to keep the temperature below 60 °C. In the next step, 1.00 g of ZrO_2_ powder was transferred to the catalyst precursor suspension for 1 h. After impregnation, the obtained catalysts were filtered and dried at 60 °C for 12 h, and then calcined in air at 500 °C for 6 h with a temperature ramp of 2 °C/min. The final catalysts were ground sieved to obtain 400–500 µm particle fractions. The catalysts were denoted by metal precursor symbols, and ZrO_2_ with an SDS suffix referring to sodium dodecyl sulphate addition at the preparation step, e.g., Pd/ZrO_2_/SDS (referring to mixed Pd precursor and zirconia with an SDS addition).

As a reference, a series of the catalysts was prepared by the incipient wetness impregnation method. The amount of the catalyst precursor (Pd(NO_3_)_2_·2H_2_O, Co(NO_3_)_2_·6H_2_O, Cr(NO_3_)_3_·9H_2_O, and Cu(NO_3_)_2_·3H_2_O), corresponding to the desired amount of metal-oxide loading, and distilled water, corresponding to incipient wetness impregnation volume, were thoroughly mixed with the oxide support. After impregnation, the obtained catalysts were filtered and dried at 60 °C for 12 h, and then calcined in air at 500 °C for 6 h with a temperature ramp of 2 °C/min. The final catalysts were ground sieved to obtain 400–500 µm particle fractions. Detailed information about catalyst preparation and characterisation is presented in Table 1, and in Table 2 for the catalysts obtained by the sonication and incipient wetness methods, respectively.

### 3.3. Catalyst Characterisation

The composition of the catalysts was determined by XRF spectroscopy (Thermo QUANT’X ED-XRF, Waltham, MA, USA). A detailed measurement description can be found elsewhere [11]. In brief, the final catalyst samples were digested in boiling nitric acid for 15 min. The catalysts’ composition was measured by using the standard calibration method. The crystalline phases of the prepared catalysts were measured by X-ray diffraction (XRD) using an Xpert’Pro diffractometer (PANalytical, Almelo, The Netherlands) with Cu Kα radiation. An analysis was carried out in the range of 5–65° 2θ with a scanning step of 0.02° 2θ. The average particle size and distribution of the sonicated catalyst precursors was determined using a dynamic light scattering (DLS Malvern Zetasizer Nano ZS, Malvern, Malvern, UK) instrument equipped with an He-Ne laser. Transmission electron microscopy analyses (TEM) were performed on JEOL JEM 2100 HT LaB6 (JEOL USA, Inc., Peabody, MA, USA). The accelerated voltage was equal 80 kV and the spot size 1 nm. Just before the TEM analyses, the catalyst samples were dropleted onto formvar film coated copper grids. The specific area and pore volumes of the prepared catalyst samples were determined by nitrogen adsorption on an ASAP 2000 volumetric adsorption system (Micromeritics Instrument Corp., Norcross, GA, USA). The morphology of the prepared samples was determined by scanning electron microscopy (SEM, FEI Company Nova Nano SEM 200 (Hillsboro, OR, USA) in backscattered electron mode. The SEM mapping experiments were performed using a JEOL 5400 scanning microscope (JEOL USA, Inc., Peabody, MA, USA) with a LINK ISIS microprobe analyser (Oxford Instruments). Prior to analysis, the catalyst samples were covered with a carbon layer. The in situ µRaman analysis was performed using a HORRIBA JobinYvon Labram1000 microscope (HORIBA Jobin Yvon IBH Ltd., Glasgow, UK) with a 532 nm laser line using a 100× objective in a Praying Mantis High Temperature Reaction Chamber (Harrick Scientific Products, Inc., Pleasantville, NY, USA). For the in situ experiments, catalyst samples were dehydrated in a He flow (50 cm^3^/min) at 450 °C for 1 h.

### 3.4. Catalytic Activity Tests

The catalysts obtained by the sonochemically aided impregnation method were tested in methane catalytic combustion. The catalytic tests were performed in a Harrick High Temperature Reaction Chamber equipped with an MKS Instruments Cirrus II LM118 (MKS Instruments, Inc., Andover, MA, USA) online quadrupole mass spectrometer. Typically, 25 mg of catalyst with a particle fraction 400–500 µm was used. During the catalytic tests, the gas composition was set to 2000 ppm CH_4_/Air (Air Products, Warszawa, Poland, calibration mixture) and controlled by Brooks 4800 mass flow controllers, resulting in a weight hourly space velocity (WHSV) equal to 150,000 cm^3^g^−1^h^−1^. Prior to the catalytic tests, the catalyst samples were dehydrated in a 50 cm^3^/min synthetic air flow (Air Products) at 450 °C for 1 h with a temperature ramp of 2 °C/min.

## 4. Conclusions

The aim of this study was to obtain and characterise non-noble metal catalysts prepared via the sonochemical method. The effect of the addition of an SDS stabiliser was investigated, and the properties of the prepared catalysts were determined by several physicochemical characterisation methods. The results obtained in this study lead to the following conclusions:

(1) There is no correlation between the addition of stabiliser and the metal loading in any of the prepared catalyst samples.
(2) The TEM analyses confirmed the decrease of particle sizes for the catalysts prepared via the sonochemical method with the addition of SDS.
(3) XRD analysis partly confirmed the presence of oxidised metal nanoparticles. The determination of the catalysts’ structure was performed by µRaman analysis. The nanoparticles are in the form of PdO, Co_3_O_4_, CuO, and Cr_2_O_3_.
(4) An SEM analysis in backscattered electron mode confirmed the presence of metal nanoparticles at the catalysts’ surfaces.
(5) An SEM/EDS mapping revealed that the active metals (Pd, Co, Cr, and Cu) are uniformly dispersed over the catalyst.
(6) The activity of the catalysts prepared via the sonochemical method is higher than the activity of their counterparts prepared by the incipient wetness method. SDS addition affects only the Pd-based catalyst, but this (about 50% of total activity) is eliminated using ultrasound irradiation.

## Figures and Tables

**Table 1:** Results of physicochemical characterisation of catalysts prepared via the sonochemical route. Abbreviation: DLS, dynamic light scattering.

Catalyst	Solution	Sonication Time, h	Metal Content, (Pd, Co, Cu, Cr), wt %	S_BET_, (m^2^/g)	V*_p_* Total (cm^3^/g)	Nanoparticle Size (DLS), nm	Specific Activity **, (mmol/gs)
Pd/ZrO_2_	0.001 M Pd(NO_3_)_2_	0.33	3.43 ± 0.89	24.23	0.14	199 ± 121	2.43 × 10^−2^
Co/ZrO_2_	0.1 M Co(NO_3_)_2_	0.33	0.020 ± 0.001	22.91	0.15	241 ± 52	2.30
Cu/ZrO_2_	0.1 M Cu(NO_3_)_2_	0.33	0.32 ± 0.02	24.76	0.15	356 ± 71	1.06 × 10^−1^
Cr/ZrO_2_	0.1 M Cr(NO_3_)_2_	0.33	0.23 ± 0.01	22.93	0.15	172 ± 22	1.71 × 10^−1^
Pd/ZrO_2_/SDS	0.001 M Pd(NO_3_)_2_	0.33	0.050 ± 0.002	25.10	0.12	324 ± 120	1.67
Co/ZrO_2_/SDS	0.1 M Co(NO_3_)_2_	0.33	2.06 ± 0.07	24.97	0.11	400 ± 131	2.69 × 10^−2^
Cu/ZrO_2_/SDS	0.1 M Cu(NO_3_)_2_	0.33	0.86 ± 0.01	22.28	0.13	201 ± 80	4.55 × ^−2^
Cr/ZrO_2_/SDS	0.1 M Cr(NO_3_)_2_	0.33	1.44 ± 0.07	23.04	0.14	188 ± 34	2.97 × 10^−2^
ZrO_2_	-	-	-	29.6, 20–30 *	0.16	-	

* Provided by the supplier; ** Specific activity at 450 °C related to the active metal loading.

**Table 2:** Results of physicochemical characterisation of reference catalysts prepared via the incipient wetness method.

Catalyst	Solution	Impregnation Time, h	Metal Content *, (Pd, Co, Cu, Cr), wt %	S_BET_, (m^2^/g)	V*_p_* Total (cm^3^/g)	Specific Activity **, (mmol/gs)
Pd/ZrO_2_/_ref_	0.001 M Pd(NO_3_)_2_	0.33	3.5	48.57	0.23	2.38 × 10^−2^
Co/ZrO_2_/_ref_	0.1 M Co(NO_3_)_2_	0.33	0.02	25.77	0.20	1.66
Cu/ZrO_2_/_ref_	0.1 M Cu(NO_3_)_2_	0.33	0.3	39.77	0.23	8.85 × 10^−2^
Cr/ZrO_2_/_ref_	0.1 M Cr(NO_3_)_2_	0.33	0.2	27.03	0.22	2.37 × 10^−1^
Pd/ZrO_2_/SDS/_ref_	0.001 M Pd(NO_3_)_2_	0.33	0.05	32.26	0.25	5.98 × 10^−1^
Co/ZrO_2_/SDS/_ref_	0.1 M Co(NO_3_)_2_	0.33	2.0	26.37	0.22	2.12 × 10^−2^
Cu/ZrO_2_/SDS/_ref_	0.1 M Cu(NO_3_)_2_	0.33	0.9	29.79	0.22	5.16 × 10^−2^
Cr/ZrO_2_/SDS/_ref_	0.1 M Cr(NO_3_)_2_	0.33	1.5	32.04	0.22	2.73 × 10^−2^

* Reference catalysts prepared via incipient wetness; ** Specific activity at 450 °C related to the active metal loading.

## Supplementary Materials

The following are available online at http://www.mdpi.com/2079-4991/7/7/174/s1, Figure S1: TEM micrographs of prepared catalysts samples; (a) ZrO_2_, (b) Pd/ZrO_2_, (c) Pd/ZrO_2_/SDS (d) Cu/ZrO_2_, (e) Cu/ZrO_2_/SDS, (f) Cr/ZrO_2_, (g) Cr/ZrO_2_/SDS, (h) Co/ZrO_2_, (i) Co/ZrO_2_/SDS; scale 200 nm; Figure S2: SEM micrographs of prepared catalysts samples; (a) Pd/ZrO_2_, (b) Pd/ZrO_2_/SDS, (c) Co/ZrO_2_, (d) Co/ZrO_2_/SDS, (e) Cu/ZrO_2_, (f) Cu/ZrO_2_/SDS, (g) Cr/ZrO_2_, (h) Cr/ZrO_2_/SDS; suffix 1 refers to coloured SEM.
