# Supplementary material for: Non-Noble Metal Oxide Catalysts for Methane Catalytic Combustion: Sonochemical Synthesis and Characterisation
Source: Nanomaterials (Basel). 2017 Jul 7;7(7):174. doi: 10.3390/nano7070174 (PMC5535240; doi:10.3390/nano7070174)
Supplement: Supplementary file 1 [file nanomaterials-07-00174-s001.pdf]

Supplementary materials

Non-noble metal oxide catalysts for methane catalytic combustion: sonochemical synthesis and characterisation

Przemysław J. Jodłowski [1,*], Roman J. Jędrzejczyk [2], Damian K. Chlebda [3], Anna Dziedzicka [1], Łukasz Kuterasiński [4], Anna Garncarczyk [5] and Maciej Sitarz [6]

[1] Faculty of Chemical Engineering and Technology, Cracow University of Technology, Warszawska 24, 31-155 Kraków, Poland; jodlowski@chemia.pk.edu.pl (PJJ); akjdz@poczta.fm (AD)
[2] Malopolska Centre of Biotechnology, Jagiellonian University, Gronostajowa 7A, 30-387 Kraków, Poland; roman.jedrzejczyk@uj.edu.pl
[3] Jagiellonian University, Faculty of Chemistry, Ingardena 3, 30-060 Kraków, Poland; damian.chlebda@uj.edu.pl
[4] Jerzy Haber Institute of Catalysis and Surface Chemistry, Polish Academy of Sciences. Niezapominajek 8, 30-239 Krakow, Poland; nckutera@cyf-kr.edu.pl
[5] Institute of Chemical Engineering, Polish Academy of Sciences, Bałtycka 5, 44-100 Gliwice, Poland; anna.g@iich.gliwice.pl
[6] Faculty of Materials Science and Ceramics, AGH University of Science and Technology, al. Mickiewicza 30, 30-059 Kraków, Poland; msitarz@agh.edu.pl

* Correspondence: jodlowski@chemia.pk.edu.pl; Tel.: +48-12-628-27-60

1. TEM particle size analysis

To determine the particle size of the support and obtained nanoparticles, TEM analyses were performed. The transmission electron microscopy analyses (TEM) were performed on JEOL JEM 2100 HT LaB6. The accelerated voltage was equal 80 kV and the spot size 1 nm. Just before the TEM analyses, the catalyst samples were dropleted onto the formvar film coated copper grids. The TEM micrographs as well as histograms of nanoparticle size analyses are presented in Figure 1S.

2. Pseudocolouring procedure

The pseudocolouring of the SEM images was previously described in [1]. The SEM and TEM images were processed using Fiji software [2]. Prior to an SEM image's evaluation, the image opened in Fiji software (File-Open…) should be converted into an 8-bit grayscale image (Image-Type-8-bit) (Figs. S2 A-H). Once the image is converted, the brightness and contrast should be adjusted to the value which enhances the visibility of the backscattered signal from the nanoparticles. The brightness and contrast adjustment is available in Image-Adjust-Brightness/Contrast. The adjustment procedure should be performed with a special care to avoid the misleading interpretation of SEM images.

The Fiji software contains preinstalled Look Up Tables (LUTs). The LUTs can be applied only for active 8-bit images. To apply LUTs for a selected image, the Image-Look up Tables (desired LUTs) have to be applied. The images with applied mpl-plasma LUTs are presented in Fig. S1 A1-H1. Once the described LUTs' colour pallete is applied to the SEM image, the image can be saved (File-Save As).

References

1. Camp, W. K.; Diaz, E.; Wawak, B.; Geologists, A. A. P.; Petroleum, B.; Ingrain, I.; Laboratories, W.; Laboratories, C. *Electron Microscopy of Shale Hydrocarbon Reservoirs: AAPG Memoir 102*; AAPG Memoir; American Association of Petroleum Geologists, 2013; ISBN 9780891813835.

2. Schindelin, J.; Arganda-Carreras, I.; Frise, E.; Kaynig, V.; Longair, M.; Pietzsch, T.; Preibisch, S.; Rueden, C.; Saalfeld, S.; Schmid, B.; Tinevez, J.-Y.; White, D. J.; Hartenstein, V.; Eliceiri, K.; Tomancak, P.; Cardona, A. Fiji: an open-source platform for biological-image analysis. *Nat Meth* **2012**, *9*, 676–682.

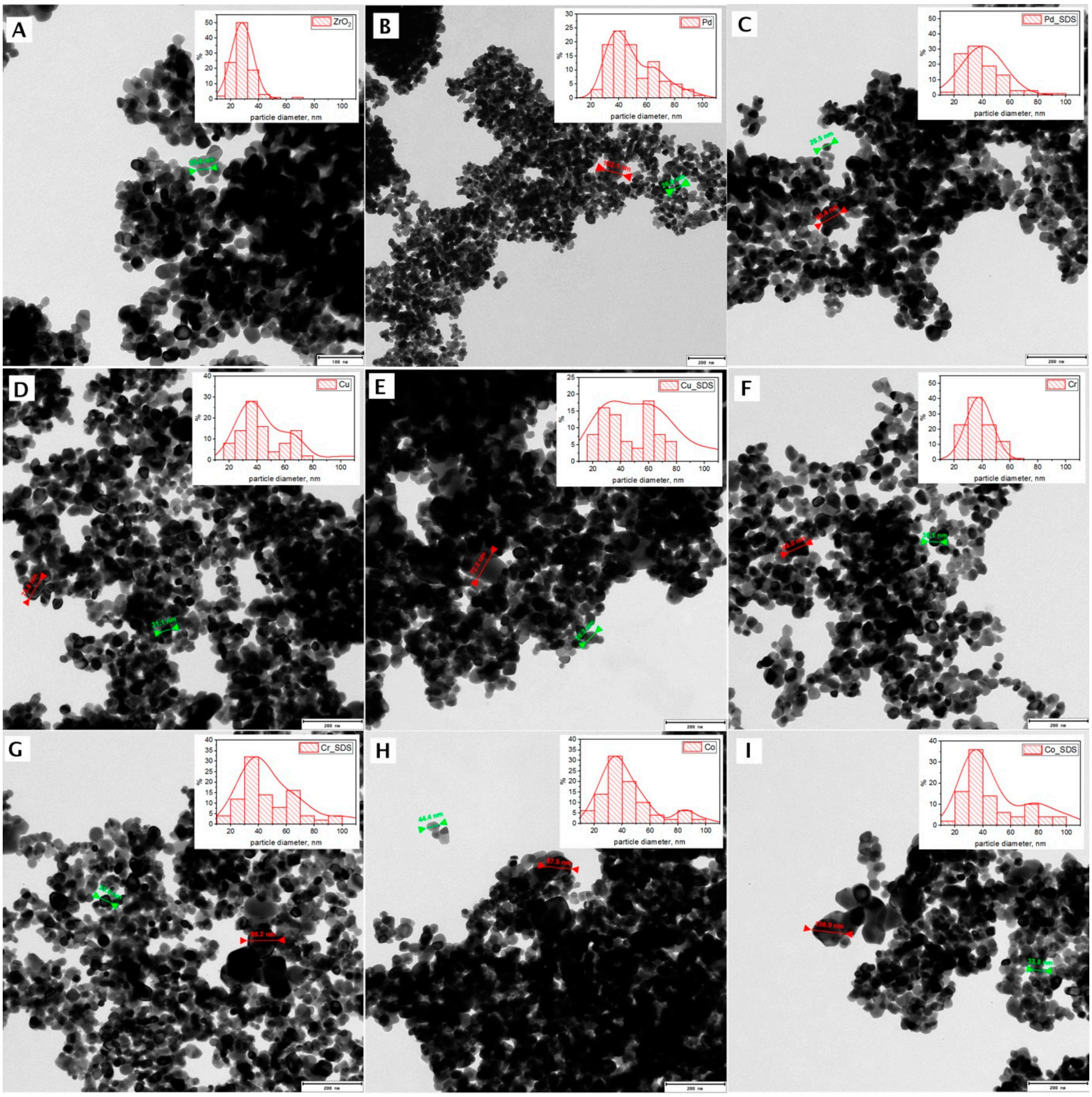

Figure S1. TEM micrographs of the prepared catalyst samples; a) ZrO_2, b) Pd/ZrO_2, c) Pd/ZrO_2/SDS d) Cu/ZrO_2, e) Cu/ZrO_2/SDS, f) Cr/ZrO_2, g) Cr/ZrO_2/SDS, h) Co/ZrO_2, i) Co/ZrO_2/SDS; scale 200 nm

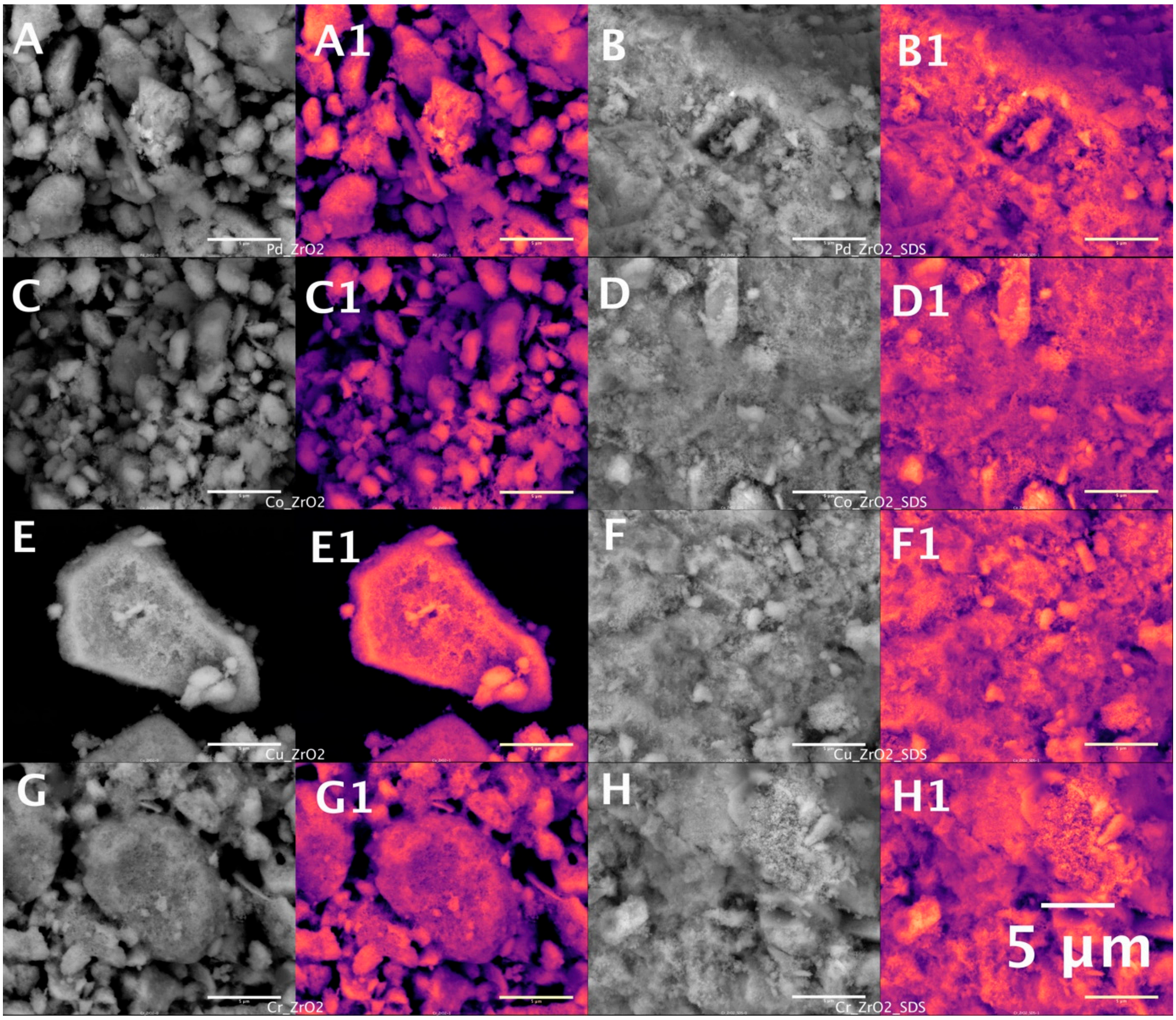

Figure S2. SEM micrographs of the prepared catalyst samples; a) Pd/ZrO_2, b) Pd/ZrO_2/SDS, c) Co/ZrO_2, d) Co/ZrO_2/SDS, e) Cu/ZrO_2, f) Cu/ZrO_2/SDS, g) Cr/ZrO_2, h) Cr/ZrO_2/SDS; suffix 1 refers to coloured SEM
